# Supplementary material for: The neurobiological basis of affect is consistent with psychological construction theory and shares a common neural basis across emotional categories
Source: Commun Biol. 2022 Dec 9;5:1354. doi: 10.1038/s42003-022-04324-6 (PMC9734184; doi:10.1038/s42003-022-04324-6)
Supplement: Supplementary file 2 — Supplementary Information [file 42003_2022_4324_MOESM2_ESM.pdf]

Supplementary Figures

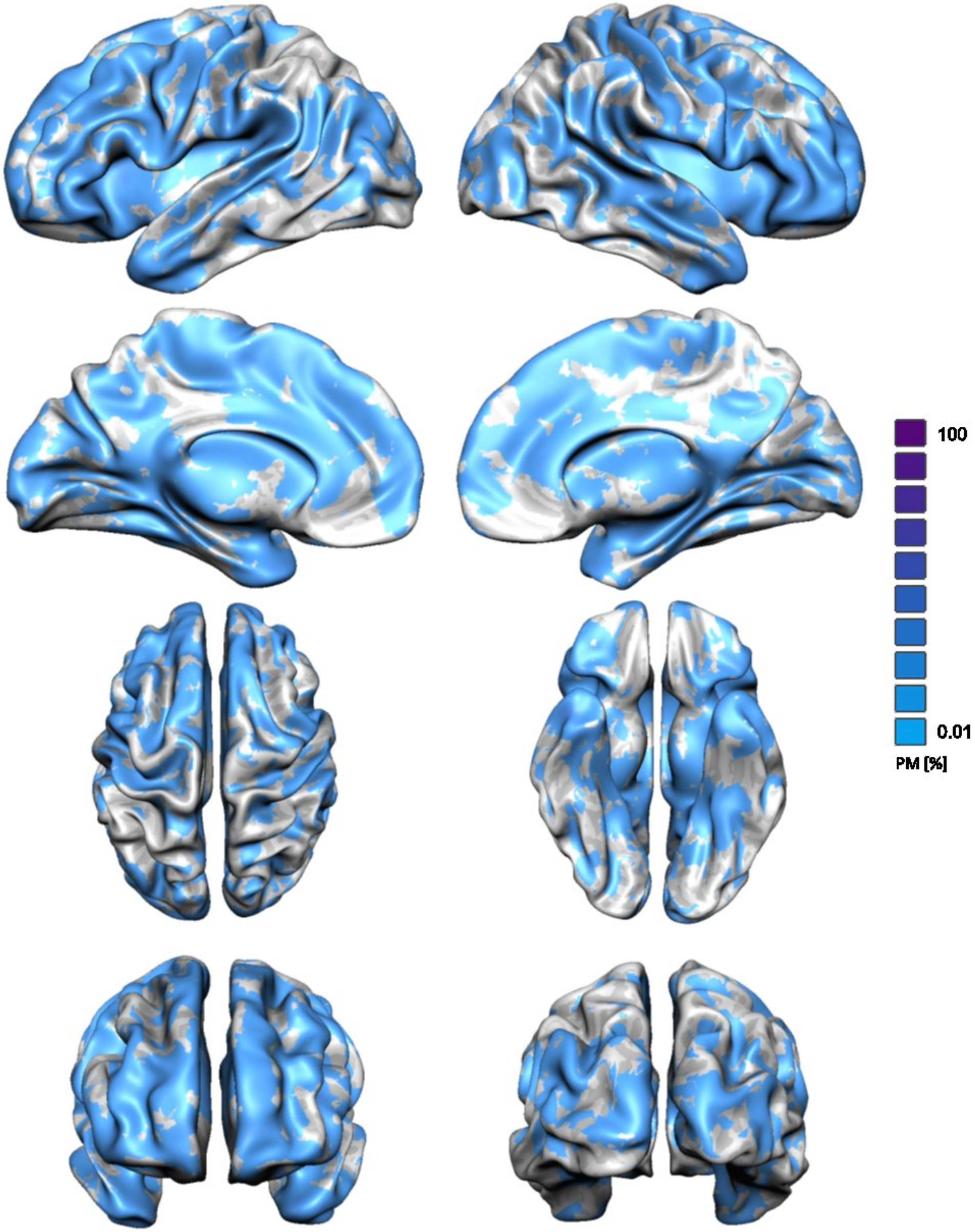

Supplementary Figure 1 Probabilistic map of spatial overlap across subjects for anger represented on an inflated cortex.

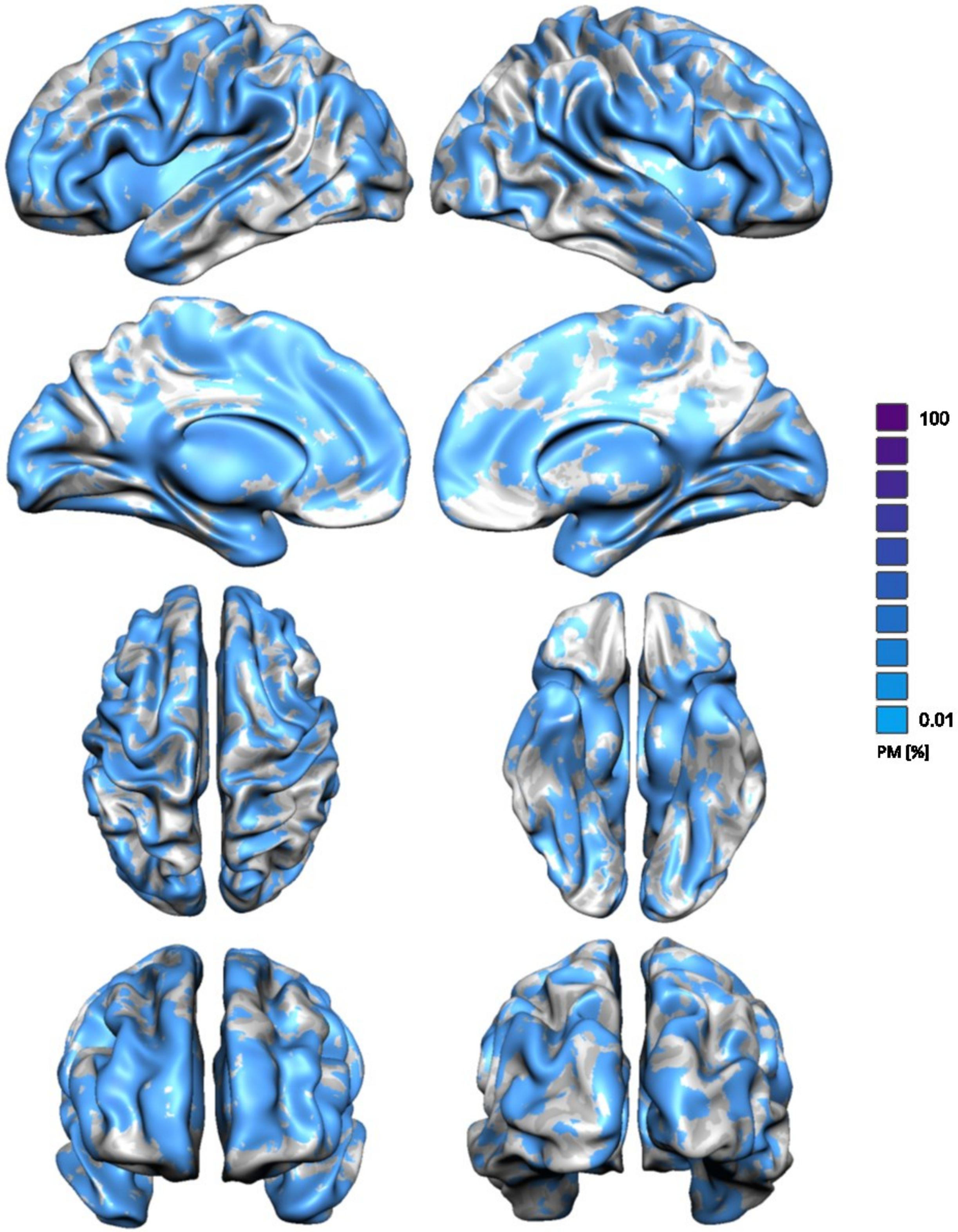

Supplementary Figure 2 Probabilistic map of spatial overlap across subjects for sadness represented on an inflated cortex.

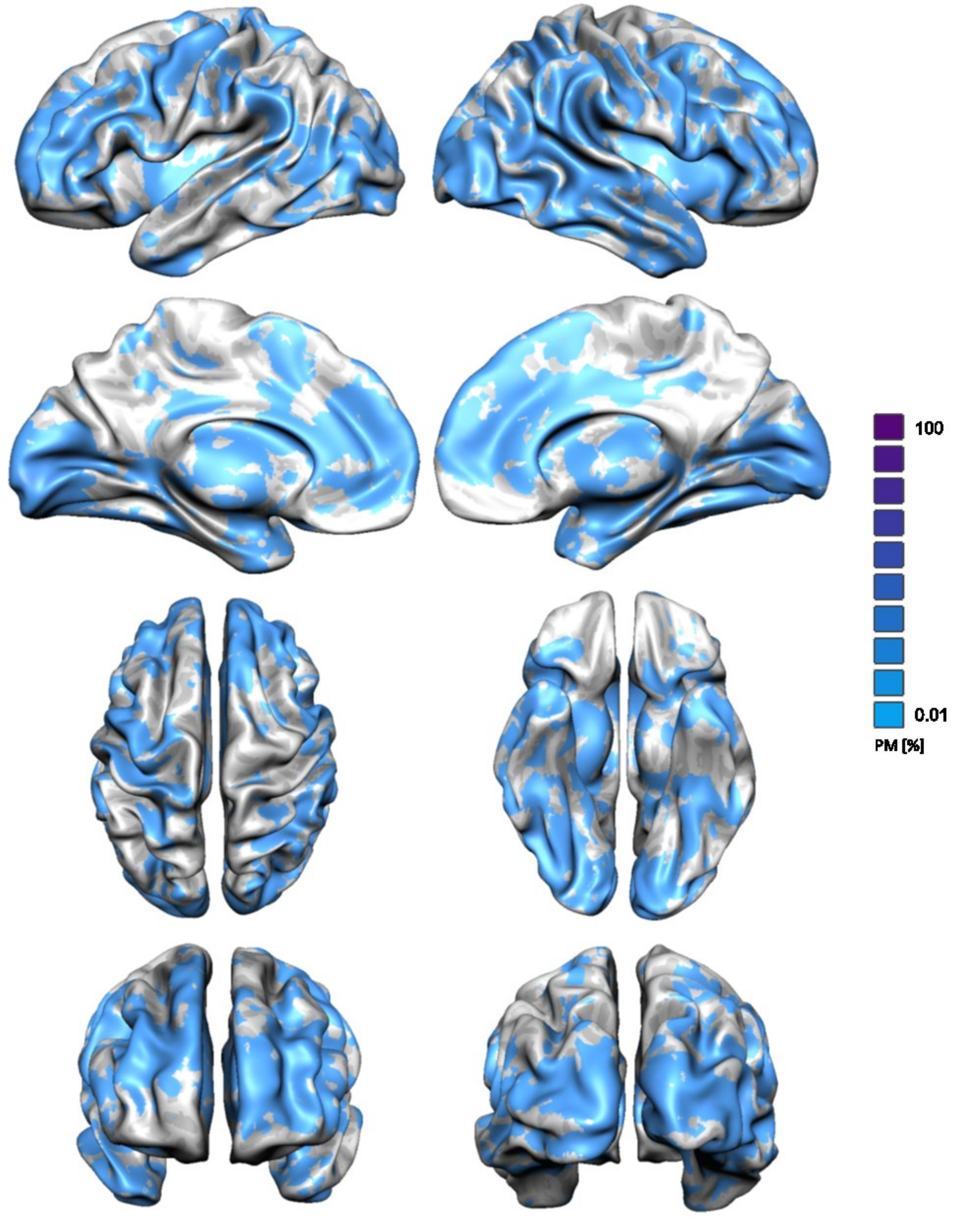

Supplementary Figure 3 Probabilistic map of spatial overlap across subjects for happiness represented on an inflated cortex.
