# Supplementary material for: The neurobiological basis of affect is consistent with psychological construction theory and shares a common neural basis across emotional categories
Source: Commun Biol. 2022 Dec 9;5:1354. doi: 10.1038/s42003-022-04324-6 (PMC9734184; doi:10.1038/s42003-022-04324-6)
Supplement: Supplementary file 5 — Reporting Summary [file 42003_2022_4324_MOESM5_ESM.pdf]

nature research

Corresponding author(s): Jan Van den Stock

Last updated by author(s): Nov 23, 2022

# Reporting Summary

Nature Research wishes to improve the reproducibility of the work that we publish. This form provides structure for consistency and transparency in reporting. For further information on Nature Research policies, see our Editorial Policies and the Editorial Policy Checklist.

## Statistics

For all statistical analyses, confirm that the following items are present in the figure legend, table legend, main text, or Methods section.

| n/a | Confirmed | |
|---|---|---|
| ☐ | ☒ | The exact sample size (*n*) for each experimental group/condition, given as a discrete number and unit of measurement |
| ☐ | ☒ | A statement on whether measurements were taken from distinct samples or whether the same sample was measured repeatedly |
| ☐ | ☒ | The statistical test(s) used AND whether they are one- or two-sided<br>*Only common tests should be described solely by name; describe more complex techniques in the Methods section.* |
| ☐ | ☒ | A description of all covariates tested |
| ☐ | ☒ | A description of any assumptions or corrections, such as tests of normality and adjustment for multiple comparisons |
| ☐ | ☒ | A full description of the statistical parameters including central tendency (e.g. means) or other basic estimates (e.g. regression coefficient) AND variation (e.g. standard deviation) or associated estimates of uncertainty (e.g. confidence intervals) |
| ☐ | ☒ | For null hypothesis testing, the test statistic (e.g. *F*, *t*, *r*) with confidence intervals, effect sizes, degrees of freedom and *P* value noted<br>*Give P values as exact values whenever suitable.* |
| ☒ | ☐ | For Bayesian analysis, information on the choice of priors and Markov chain Monte Carlo settings |
| ☒ | ☐ | For hierarchical and complex designs, identification of the appropriate level for tests and full reporting of outcomes |
| ☐ | ☒ | Estimates of effect sizes (e.g. Cohen's *d*, Pearson's *r*), indicating how they were calculated |

*Our web collection on statistics for biologists contains articles on many of the points above.*

## Software and code

Policy information about availability of computer code

| | |
|---|---|
| Data collection | No software was used |
| Data analysis | - Imaging data were analyzed using BrainVoyager 22.0 (Goebel, R. BrainVoyager--past, present, future. Neuroimage 62, 748-756, 618 doi:10.1016/j.neuroimage.2012.01.083 (2012)) and Neuroelf v1.1 (http://neuroelf.net) within MatLab R2020b (Mathworks, Inc).<br>- RStudio Desktop 2022.07.1+554 (RStudio: Integrated Development for R. RStudio, PBC (Boston, MA, 2022)) within R 4.2.1 (R: A language and environment for statistical computing (R Foundation for Statistical 621 Computing, Vienna, Austria, 2022)) was used for statistical analysis. |

For manuscripts utilizing custom algorithms or software that are central to the research but not yet described in published literature, software must be made available to editors and reviewers. We strongly encourage code deposition in a community repository (e.g. GitHub). See the Nature Research guidelines for submitting code & software for further information.

## Data

Policy information about availability of data

All manuscripts must include a data availability statement. This statement should provide the following information, where applicable:

- Accession codes, unique identifiers, or web links for publicly available datasets
- A list of figures that have associated raw data
- A description of any restrictions on data availability

The data that support the findings of this study are available from the corresponding author, upon request. Data are still being analyzed for other purposes and cannot be made publicly available at this time. The source data underlying the graphs are provided as Supplementary Data 1.

# Field-specific reporting

Please select the one below that is the best fit for your research. If you are not sure, read the appropriate sections before making your selection.

☐ Life sciences ☒ Behavioural & social sciences ☐ Ecological, evolutionary & environmental sciences

For a reference copy of the document with all sections, see nature.com/documents/nr-reporting-summary-flat.pdf

# Behavioural & social sciences study design

All studies must disclose on these points even when the disclosure is negative.

| | |
|---|---|
| Study description | Quantitative methods |
| Research sample | 37 healthy subjects (19 females, mean age= 37 years, SD age= 12.656 years) were participated in our study. |
| Sampling strategy | Participants were recruited via our database and public advertisements. |
| Data collection | The words relating to emotionally intense autobiographical experiences that are provided by the subjects prior to imaging sessions were displayed on the screen within the scanner. Brain imaging was performed using a 3 Tesla (MR) scanner 396 (Achieva3T; Philips, Best, the Netherlands) with a 32-channel head coil.<br>Only the researcher and the participant were present. |
| Timing | First inclusion fMRI: 23.07.2012<br>Last inclusion fMRI: 11.07.2014 |
| Data exclusions | Both runs of one participant and one run of three participants were excluded due to excessive motion in the scanner (>3 mm). One run of one participant was excluded due to inaccurate understanding of the instruction. This resulted in 36 participants with at least 1 run and 32 participants of which both runs were included. |
| Non-participation | No drop-out |
| Randomization | Participants were not allocated into experimental groups. |

# Reporting for specific materials, systems and methods

We require information from authors about some types of materials, experimental systems and methods used in many studies. Here, indicate whether each material, system or method listed is relevant to your study. If you are not sure if a list item applies to your research, read the appropriate section before selecting a response.

## Materials & experimental systems

| n/a | Involved in the study | |
|---|---|---|
| ☒ | ☐ | Antibodies |
| ☒ | ☐ | Eukaryotic cell lines |
| ☒ | ☐ | Palaeontology and archaeology |
| ☒ | ☐ | Animals and other organisms |
| ☐ | ☒ | Human research participants |
| ☒ | ☐ | Clinical data |
| ☒ | ☐ | Dual use research of concern |

## Methods

| n/a | Involved in the study | |
|---|---|---|
| ☒ | ☐ | ChIP-seq |
| ☒ | ☐ | Flow cytometry |
| ☐ | ☒ | MRI-based neuroimaging |

## Human research participants

Policy information about studies involving human research participants

| | |
|---|---|
| Population characteristics | See above |
| Recruitment | Participants were recruited via our database and public advertisements. |
| Ethics oversight | The study was approved by the Ethical Committee of University Hospitals Leuven (ML8040) and written informed consent of the participants was obtained according to the Declaration of Helsinki. |

Note that full information on the approval of the study protocol must also be provided in the manuscript.

# Magnetic resonance imaging

## Experimental design

| | |
|---|---|
| Design type | Block design |
| Design specifications | At least two weeks prior to the brain imaging session, participants were instructed to select six autobiographical events that were associated with intense emotional experiences: two angry, two happy, and two sad events. Furthermore, they were instructed to think of two emotionally neutral autobiographical events. For each event, they were asked to provide a single word that would unambiguously be associated with the specific event. The imaging experiment consisted of two functional runs, each containing one event of each emotion category. A run started with a 1000 ms presentation of a black screen, followed by presentation of one of the provided words for 3000 ms. Subsequently, “Close your eyes now” was displayed for 2000 ms. During the following 61 s, a black screen was presented, which constituted the emotion experience block. Prior to the scanning, participants were instructed to try to re-experience the respective emotion during this block as intensely as possible. The end of this block was signaled to the participant by three alternating 500 ms presentations of black and white screens, which were easily detectable with the eyes closed. Subsequently, an emotion intensity rating was performed by means of a visual analogue scale. This event consisted of a 10s presentation of a slider on which participants could rate how intensely they had re-experienced the emotion (from ‘very weak’ to ‘very intense’). After this interval, the text “Press after vertical line” was displayed for 4000 ms. Subsequently, 30 stimuli consisting of a circle filled with line gratings were randomly presented one by one for 500 ms each with a 500 ms inter-stimulus interval. Five stimuli displayed vertical gratings and 25 displayed horizontal gratings. This cognitively demanding visual reaction time task was included to minimize emotional carry-over effects between emotion categories. Subsequently, this procedure was repeated 3 times within a run, albeit each with a different provided word presented. The order of emotion categories was counterbalanced. The emotion experience blocks were followed by the visual reaction time task to minimize emotional carry-over effects. For two of the participants, the duration of the experimental procedure was slightly different due to 1000 ms instead of 500 ms presentations of the line grating stimuli. A high-resolution structural scan was performed in between both functional runs. |
| Behavioral performance measures | The subjective emotion intensities were recorded via a visual analogue scale within the scanner. The participants rated how intensely they had re-experienced the emotion from ’very weak’ to ’very intense’ on a scale of 1 to 10. First, we tested whether the intensity ratings averaged over events were normally distributed using Shapiro-Wilk tests. Depending on the resulting normality, the differences in intensity ratings between emotion categories were evaluated by means of parametric or non-parametric testing. Furthermore, we tested whether the intensity ratings across subjects per event were normally distributed using Shapiro-Wilk tests. Then again depending on the normality, we tested whether the intensity ratings of two events of the same emotion category differed using parametric or non-parametric paired testing. |

## Acquisition

| | |
|---|---|
| Imaging type(s) | Structural and functional MRI |
| Field strength | 3 Tesla |
| Sequence & imaging parameters | The fMRI was performed using a 3 Tesla (MR) scanner 396 (Achieva3T; Philips, Best, the Netherlands) with a 32-channel head coil. Functional runs had a duration of 507 s each (532 s for the two participants with a slightly different protocol). 140 T2*-weighted Blood-Oxygenation Level Dependent (BOLD) contrast volumes were acquired (153 and 150 volumes for the two deviant participants). A functional volume consisted of 70 axial slices oriented parallel to the anterior commissure — posterior commissure (AC-PC) plane with 2.0 mm slice thickness, no gap, 2.75 x 2.75 mm in-plane resolution, 80 x 80 matrix size, and 220 x 220 mm field of view (FOV) and covered the whole brain. The echo time (TE) (26 ms), the repetition time (TR) (3500 ms) and flip angle (90°) were optimized for subcortical sensitivity. The first four volumes were dummy volumes to allow for T1 equilibration. In between the functional runs, a high resolution T1-weighted anatomical image with 1 x 1 x 1 mm voxel size was acquired by using a three-dimensional (3-D) magnetization-prepared-rapid acquisition gradient echo sequence as 182 slices with 4.6 ms of TE, 9.6 ms of TR, 256 x 256 matrix size. |
| Area of acquisition | Whole brain scan |

Diffusion MRI ☐ Used ☒ Not used

## Preprocessing

| | |
|---|---|
| Preprocessing software | Imaging data were preprocessed using BrainVoyager 22. Pre-processing included realignment for motion correction, slice time correction, coregistration of the anatomical and functional images, spatial normalization of both anatomical and functional images to Montreal Neurological Institute (MNI) standard space with 2 mm x 2 mm x 2 mm voxel size, and smoothing of the images using a Gaussian kernel of 8 mm full width at half maximum (FWHM) as final step. |
| Normalization | Both anatomical and functional images underwent spatial normalization to MNI standard space with 2 mm x 2 mm x 2 mm voxel size |
| Normalization template | Montreal Neurological Institute (MNI) standard space with 2 mm x 2 mm x 2 mm voxel size |
| Noise and artifact removal | Realignment for motion correction, slice time correction, coregistration of the anatomical and functional images, spatial |

Noise and artifact removal

normalization of both anatomical and functional images to MNI standard space with 2 mm x 2 mm x 2 mm voxel size, and smoothing of the images using a Gaussian kernel of 8 mm FWHM as final step.

Volume censoring

Imaging data were preprocessed using BrainVoyager 22. Pre-processing included realignment for motion correction, slice time correction, coregistration of the anatomical and functional images, spatial normalization of both anatomical and functional images to Montreal Neurological Institute (MNI) standard space with 2 mm x 2 mm x 2 mm voxel size, and smoothing of the images using a Gaussian kernel of 8 mm full width at half maximum (FWHM) as final step.

## Statistical modeling & inference

Model type and settings

A first level general linear model (GLM) was constructed with 9 regressors defined by the onset and offset of the 4 emotion experience epochs, the 4 visual reaction time epochs, and a constant, convolved with the canonical hemodynamic response function. The entire 61 seconds of emotion experience event was modelled as single event and the intensity ratings given by the subjects were included as weights in the GLM. Next, a random effects (RFX) group GLM analysis was performed using a second-order autoregressive model for removal of serial correlations and time course normalization using z transformation.

Effect(s) tested

Emotion-specific activation was investigated by contrasting each emotion category with each of the 3 other categories via voxel-wise whole brain conjunction analyses, e.g. for anger: anger>sad ∩ anger>happy ∩ anger>neutral. Furthermore, we calculated the beta-values (averaged over voxels) for anger vs each of the other categories, sadness vs each of the other categories, and happiness vs each of the other categories in the OFC, MPFC, and ACC ROIs, respectively.
Regional overlap in activation between events of a single emotion category was investigated by means of voxel-wise whole-brain within emotion category between events conjunction analyses. We contrasted each emotion category with the within run neutral condition and subsequently ran 3 conjunction analyses, one for each of the 3 contrasts (e.g. conjunction of anger vs neutral in the first run with anger vs neutral in the second run), resulting in three statistical overlap maps, one for each emotion category.
To investigate the similarity of neural patterns within emotion categories (vs neutral) across subjects, we performed similarity analyses over events and compared the Pearson correlation coefficients between different events of the same emotion category to Pearson correlation coefficients between events of different emotion categories.
Further, to investigate spatial overlap between subjects within emotion categories, we first identified for each subject the regions that were active during each emotion category and subsequently created binary maps, of which we calculated the percentage overlap across subjects for each emotion category.
Next, to investigate whether the pattern similarity across subjects within an emotion category is higher than between emotion category, we compared pairwise Pearson correlation coefficients between subjects within emotion category to pairwise Pearson correlation coefficients between subjects between emotion category
We performed 6 voxel-wise whole-brain conjunction analyses between activation for each of the three emotion category pairs (anger-sad; anger-happy; anger-neutral; sad-happy; sad-neutral; happy-neutral). Each of the emotion conditions was compared to implicit baseline and not to the neutral condition.
We also investigated whether there were associations between emotion categories in the regional activation level across subjects by calculating voxel-wise whole-brain Pearson correlation coefficients between each of the sixemotion category pairs (anger-sad; anger-happy; anger-neutral; sad-happy; sad-neutral; happy-neutral).
Finally, to investigate consistency between emotion categories (vs baseline) across subjects, we performed similarity analyses over events and compared the Pearson correlation coefficients between different events of the same emotion category to Pearson correlation coefficients between events of different emotion categories.

Specify type of analysis: ☐ Whole brain ☐ ROI-based ☒ Both

Anatomical location(s)

First, we used an independent dataset to define a general affect network (GAN). This was based on the results of a meta-analysis of 397 neuroimaging studies of emotion experience and perception (Lindquist, K. A., Satpute, A. B., Wager, T. D., Weber, J. & Barrett, L. F. The Brain Basis of Positive and Negative Affect: Evidence from a Meta-Analysis of the Human Neuroimaging Literature. Cereb Cortex 26, 1910-1922, doi:10.1093/cercor/bhv001 (2016)). In particular, the GAN was defined by combining the meta-analytic maps for negative and positive affect. We used an inclusive approach and defined the GAN as the pooled clusters of each map, using a lenient threshold of 0.06 on each proportion map. Furthermore, we combined a global and local approach in addressing a priori associations between emotion category and brain region. The global approach consisted of anatomical definition the entire OFC and ACC using the 'automated anatomical labelling' atlas (Tzourio-Mazoyer, N. et al. Automated anatomical labeling of activations in SPM using a macroscopic anatomical parcellation of the MNI MRI single-subject brain. Neuroimage 15, 273-289, doi:10.1006/nimg.2001.0978 (2002)) and the local approach of defining 6 mm radius spherical ROIs centred around peak coordinates that show consistent responses to anger, sadness, and happiness, based on meta-analytic findings respectively in the OFC, MPFC and ACC (Vytal, K. & Hamann, S. Neuroimaging support for discrete neural correlates of basic emotions: a voxel-based meta-analysis. J Cogn Neurosci 22, 2864-2885, doi:10.1162/jocn.2009.21366 (2010)).

Statistic type for inference
(See Eklund et al. 2016)

The whole brain voxel-wise statistical threshold was set at p<.05 FDR-corrected combined with a maximal uncorrected p-value of .001. Minimal cluster size was established via 1000 Monte Carlo simulations of random image generation, followed by the injection of spatial correlations between neighboring voxels, voxel intensity thresholding, and cluster identification.

Correction

The whole brain voxel-wise statistical threshold was set at p<.05 FDR-corrected combined with a maximal uncorrected p-value of .001. Minimal cluster size was established via 1000 Monte Carlo simulations of random image generation, followed by the injection of spatial correlations between neighboring voxels, voxel intensity thresholding, and cluster identification.

## Models & analysis

| n/a | Involved in the study |
|---|---|
| ☒ | ☐ Functional and/or effective connectivity |
| ☒ | ☐ Graph analysis |
| ☐ | ☒ Multivariate modeling or predictive analysis |

Multivariate modeling and predictive analysis

- We performed similarity analyses between 1 of the 2 events of each emotion category and the remaining events of each emotion category (e.g. similarity analyses between anger vs neutral in the first run and anger vs neutral in the second run, between anger vs neutral in the first run and sadness vs neutral in the second run). In order to investigate whether the pattern similarity across events within an emotion category is higher than between emotion category, for each emotion category, pairwise Fisher Z-transformed Pearson correlation coefficients between events within emotion category were compared to pairwise Fisher Z-transformed Pearson correlation coefficient between events of that emotion category and events of other emotion categories (e.g. whether the correlations between anger vs neutral in the first run and anger vs neutral in the second run is significantly higher than correlations between anger vs neutral in the first run and sadness vs neutral in the second run) in the GAN. First, the normality of the distribution of pairwise Fisher Z-transformed Pearson correlation coefficients between events was tested for each variable individually, pooled within emotion correlations, and pooled between emotion correlations by means of Shapiro-Wilk tests. Depending on the results, the assessment of the significance was performed by means of 2 parametric or non-parametric paired testing on sets of 32 within subject between event Fisher Z-transformed correlations for each emotion category separately and by parametric or non-parametric testing on pooled within emotion correlations on the one hand and pooled between emotion correlations on the other hand.

-We performed similarity analyses across subjects for within each emotion category and between each emotion category pair, revealing all 630 (for each within emotion category) and 1260 (for each between emotion) pairwise between subject Pearson correlations of the neural patterns in the GANof each ROI37. In order to investigate whether the pattern similarity across subjects within an emotion category is higher than between emotion category, we compared pairwise Fisher Z-transformed Pearson correlation coefficients between subjects within emotion category to pairwise Fisher Z-transformed Pearson correlation coefficients between subjects between emotion category for each emotion category separately and all emotion categories combined (pooled within emotion categories vs pooled between emotion categories). First, Tthe normality of the distribution of all 630 pairwise Fisher Z- transformed Pearson correlation coefficients was tested using Shapiro-Wilk tests for each variable individually, pooled within emotion correlations, and pooled between emotion correlations. emotion category. Depending on the normality, the assessment of pattern similarity significance was performed by means of 2 parametric or non-parametric paired testing on all 630 pairwise Fisher Z-transformed Pearson correlation coefficients for every emotion category separately and by parametric or non-parametric testing on pooled within emotion correlations and pooled between emotion categories.

- Similarity analyses between events were performed exactly in the same approach as explained above but emotion conditions were compared to implicit baseline instead of to the neutral condition. In order to investigate whether within emotion between event correlations are different than the between emotion category between event correlations, the assessment of the significance was performed again as explained above.
